# Implications of GCLC in prognosis and immunity of lung adenocarcinoma and multi-omics regulation mechanisms

**DOI:** 10.1186/s12890-024-03052-3

**Published:** 2024-05-15

**Authors:** Zhong Huang, Feifei Liang, Jiangtao Wu, Zichong Huang, Yinglian Li, Xiaoyuan Huang, Zhenyu Liu

**Affiliations:** 1 Department of Oncology, KaiYuan Langdong Hospital of Guangxi Medical University, Nanning, Guangxi 530028 China; 2 Department of Radiation Oncology, The First Affiliated Hospital of Guangxi Medical University, Nanning, Guangxi 530021 China

**Keywords:** GCLC, Lung adenocarcinoma, Prognosis, Model, Immunity, Ferroptosis, Multi-omics

## Abstract

**Background:**

Ferroptosis is an iron-dependent type of regulated cell death, and has been implicated in lung adenocarcinoma (LUAD). Evidence has proved the key role of glutamate-cysteine ligase catalytic subunit (GCLC) in ferroptosis, but its role in LUAD remains unclear. Herein, we explored the implications of GCLC and relevant genes in LUAD prognosis and immunity as well as underlying molecular mechanisms.

**Methods:**

This work gathered mRNA, miRNA, DNA methylation, somatic mutation and copy-number variation data from TCGA-LUAD. WGCNA was utilized for selecting GCLC-relevant genes, and a GCLC-relevant prognostic signature was built by uni- and multivariate-cox regression analyses. Immune compositions were estimated via CIBERSORT, and two immunotherapy cohorts of solid tumors were analyzed. Multi-omics regulatory mechanisms were finally assessed.

**Results:**

Our results showed that GCLC was overexpressed in LUAD, and potentially resulted in undesirable survival. A prognostic model was generated, which owned accurate and independent performance in prognostication. GCLC, and relevant genes were notably connected with immune compositions and immune checkpoints. High GCLC expression was linked with better responses to anti-PD-L1 and anti-CTLA-4 treatment. Their possible DNA methylation sites were inferred, e.g., hypomethylation in cg19740353 might contribute to GCLC up-regulation. Frequent genetic mutations also affected their expression. Upstream transcription factors (E2F1/3/4, etc.), post-transcriptional regulation of miRNAs (hsa-mir-30c-1, etc.), lncRNAs (C8orf34-AS1, etc.), and IGF2BP1-mediated m^6^A modification were identified. It was also found NOP58-mediated SUMOylation post-translational modification.

**Conclusions:**

Together, we show that GCLC and relevant genes exert crucial roles in LUAD prognosis and immunity, and their expression can be controlled by complex multi-omics mechanisms.

**Supplementary Information:**

The online version contains supplementary material available at 10.1186/s12890-024-03052-3.

## Background

Lung cancer remains a dominating cause of cancer-related deaths globally, with lung adenocarcinoma (LUAD) as the most prevalent subtype, occupying nearly half of all cases [1]. The incidence of LUAD is increasing in most countries, notably among women and nonsmokers [2]. LUAD is mostly diagnosed at advanced stages, thus limiting therapeutic options (surgical resection, chemoradiotherapy, targeted therapy and immunotherapy) [3]. The five-year survival remains <15% [4]. Altogether, in-depth comprehending of molecular mechanisms underlying LUAD is urgently required.

LUAD is highly heterogeneous and consists of malignant cells with diverse histological subtypes [5]. The genetic, epigenetic and transcriptomic features may result in LUAD heterogeneity [6]. Ferroptosis represents an iron-dependent form of regulated cell death, which is attributed to the superfluous build-up of lipid peroxides on cellular membrane [7]. This cell death has been found to be connected with LUAD. For instance, targeting histone deacetylase heightens the treatment effects of Erastin-driven ferroptosis in EGFR-mutant LUAD [8]. Endogenous glutamate can determine ferroptosis sensitivity through ADCY10-mediated YAP inhibition in LUAD [9]. METTL3 results in LUAD tumor growth as well as mitigates ferroptosis through stabilizing SLC7A11 m^6^A modification [10]. Cysteine is required for maintaining cellular redox homeostasis both in normal and transformed cells, and cysteine deficiency can result in ferroptosis [11]. Cystine starvation in non‐small‐cell lung cancer cells causes the accumulation of gamma-glutamine peptides owing to the non-canonical activity in glutamate-cysteine ligase catalytic subunit (GCLC). In addition, GCLC exerts a glutathione-independent, non-canonical function in protecting against ferroptosis through maintaining glutamate homeostasis under cystine deficiency [12]. However, the role of GCLC in LUAD is largely unknown [13, 14]. To solve the problem, in this work, we comprehensively assessed the implications of GCLC and relevant genes in LUAD prognosis and immunity and explored potential multi-omics regulatory mechanisms. Our findings uncovered that GCLC was overexpressed in LUAD, and linked with poor prognostic outcomes. In addition, high GCLC expression predicted better responses to anti-PD-L1 and anti-CTLA-4 treatment. Aberrant expression of GCLC and relevant genes was potentially controlled by complex multi-omics regulatory mechanisms. Altogether, GCLC was identified as a possible prognostic biomarker and therapeutic target for LUAD.

## Materials and methods

### Acquisition of LUAD datasets

RNA sequencing profiles of LUAD (*n=*525) and normal (*n=*60) tissues were retrieved from The Cancer Genome Atlas (TCGA) (https://portal.gdc.cancer.gov). In addition, matched clinical data were also gathered. Supplementary table 1 listed the details of patient clinical characteristics. After normalization, transcriptome data were utilized for subsequent analysis.

### Differential expression analysis

The evaluation of genes with differential expression between LUAD versus controls and between lowly versus highly expressed GCLC LUAD was carried out by use of limma package [15]. The threshold was set as adjusted *p*<0.05. The shared DEGs were utilized for subsequent analyses.

### Weighted correlation network analysis (WGCNA)

Co-expression modules were established through WGCNA package [16]. The appropriate soft thresholding power was determined by pickSoftThreshold function. Genes with high connection were merged into distinct co-expression modules via hierarchical clustering along with dynamic tree cut approaches. Co-expression module structure was visualized by a heatmap plot of gene inter-connectivity utilizing TOMplot function. The eigengene network was built through dendrogram along with a heatmap plot based upon labeledHeatmap function. Module eigengene (ME), the first principal component of one module, which can represent the gene expression profiling in the module. Pearson’s test was implemented on the MEs of the merged modules with clinical features. Module membership (MM) is denoted as the correlation of gene expression profiling with the ME of one module, while gene significance (GS) represents the connection between gene expression profiling and clinical features. The relationships of the two were assessed for further selection of intramodular genes.

### Functional enrichment analysis

Through adopting clusterProfiler approach [17], Kyoto Encyclopedia of Genes and Genomes (KEGG) pathways enriched by module genes were probed. In addition, the enrichment on Gene Ontology (GO) was implemented. Terms with adjusted *p*<0.05 denoted the significant enrichment.

### Prognostic model construction

Brown module genes were included in the univariate-cox regression analysis. Those with *p*<0.01 were adopted for the establishment of a multivariate-cox regression model. The formula included: RiskScore = $\sum\nolimits_{1}^{n}\mathrm{coefficient }\,\left({\text{beta}}\right)\, of\, gene\, (i)*expression\, of \,gene \,(i)$. TCGA-LUAD samples were randomized into the discovery and test sets. Based upon the optimal cutoff, low- and high-RiskScore groups were classified. Afterwards, survival outcomes were compared between groups. Pearson’s test on RiskScore and GCLC with clinical parameters was also implemented. Uni- and multivariate-cox regression analyses were carried out on RiskScore and clinical traits with LUAD survival. A nomogram was generated by use of rms package, which integrated RiskScore and common clinicopathologic variables. Calibration curves were utilized for the evaluation of prediction efficacy of this model.

### Tumor-infiltrating immune cell estimation

Immune compositions in bulk tissues were estimated by use of CIBERSORT approach based upon the integration of support vector regression and expression profiling from purified leukocyte gene sets [18].

### DNA methylation analysis

DNA methylation data (Illumina Human Methylation 450) of LUAD specimens were downloaded from TCGA database. The SMART tool was adopted for comprehensively analyzing and visualizing DNA methylation data, as previously described [19]. Difference in DNA methylation probe levels was compared between groups, and the influence of DNA methylation levels on gene expression was assessed through correlation analysis.

### Genetic mutation evaluation

The somatic variants (Mutation Annotation Format) from TCGA database were estimated based upon maftools package [20]. Copy number alterations of LUAD acquired from TCGA database were preprocessed utilizing GISTIC2.0 algorithm [21].

### Post-transcriptional modulation by non-coding RNAs

MicroRNA (miRNA) expression profiles of LUAD were retrieved from TCGA database. MiRNAs with differential expression were selected between controls and LUAD or between lowly and highly expressed GCLC LUAD specimens utilizing limma package under the cutoff of adjusted *p*<0.05. Interactions of the shared miRNAs and long non-coding RNAs (lncRNAs) with GCLC and relevant prognostic genes were then assessed.

### Analysis of transcription factors, N^6^-methyladenosine (m^6^A) regulators and SUMOylation regulators

Correlation analysis on transcription factors, m^6^A and SUMOylation regulators with GCLC and relevant prognostic genes was also carried out across LUAD specimens.

### Statistical analysis

R packages (version 4.0.2) were adopted for statistical analysis. Comparison between two groups was implemented by use of student’s t or Wilcoxon test, with One-way ANOVA or Kruskal-Wallis test for comparison between three groups. Kaplan-Meier curves were plotted on overall survival (OS), with log-rank test for survival difference. Uni- and multivariate-cox regression models were also utilized for survival analysis. Correlation analysis was implemented through Pearson’s or Spearman’s test. Two-sided *p*<0.05 denoted statistical significance.

## Results

### GCLC is overexpressed in LUAD and linked with patient prognosis

**Fig. 1:**
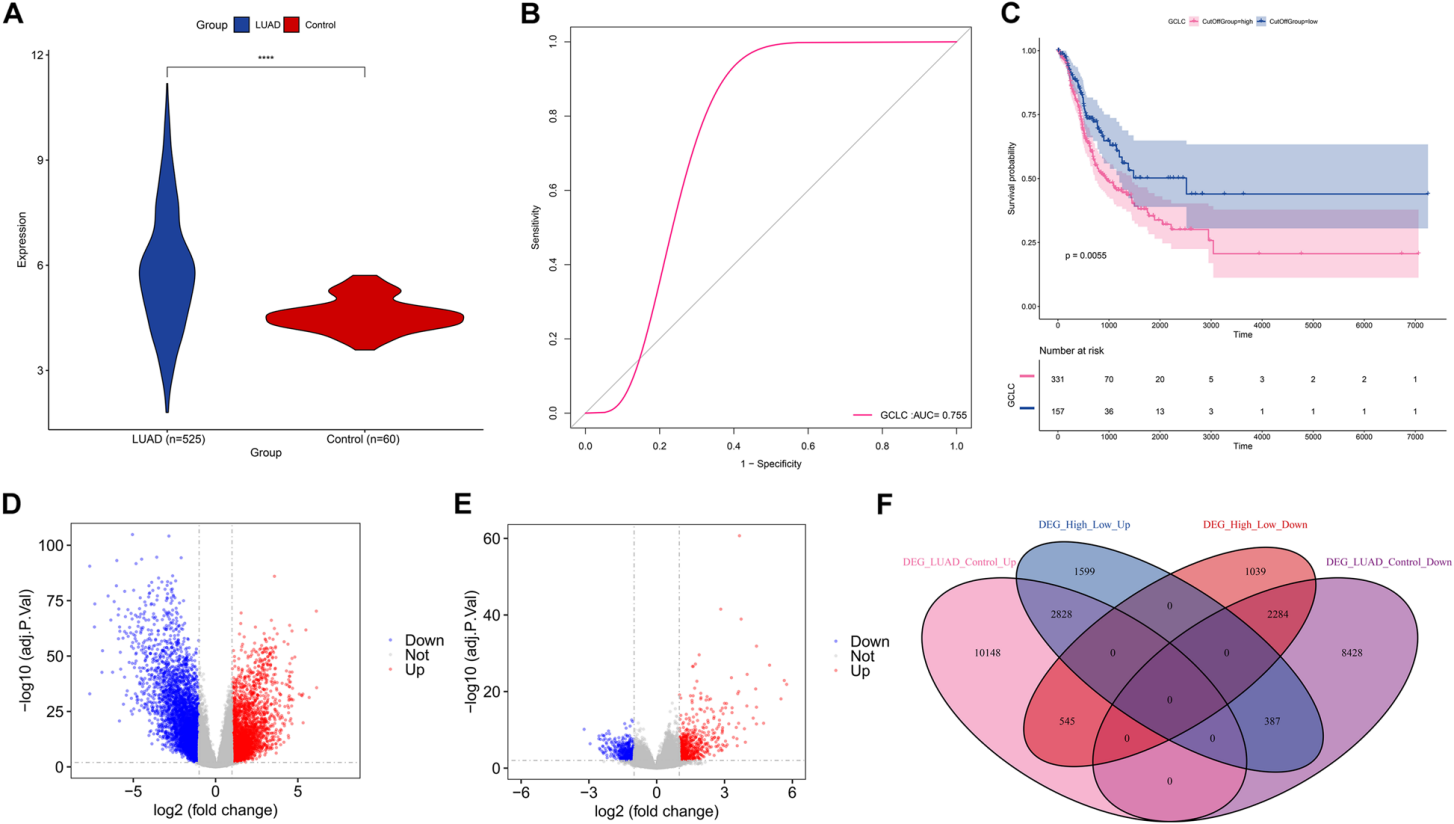
Evaluation of the deregulation and survival relevance of GCLC in LUAD and selection of GCLC-related DEGs. **A** Comparison of GCLC expression in LUAD (*n=*525) and control (*n=*60) tissue specimens. **B** ROC of GCLC in distinguishing LUAD from controls. **C** Survival probability of patients with lowly (*n=*157) or highly (*n=*331) expressed GCLC. Survival difference was evaluated via log rank test. **D** Volcano plot of DEGs in LUAD versus control tissues. **E** Volcano plot of DEGs between lowly and highly expressed GCLC LUAD samples. **F** Venn diagram illustrating the shared DEGs between above two gene sets. Student’s t test was used for comparison between two groups. ****p<0.0001

Our results showed that GCLC was frequently overexpressed in LUAD relative to control specimens in TCGA-LUAD cohort (Fig. 1A). It was also proven that GCLC was capable of well differentiating LUAD from controls (Fig. 1B). Next, we evaluated survival significance of GCLC in LUAD. As illustrated in Fig. 1C, LUAD patients with lowly expressed GCLC owned the remarkable survival superiority in comparison to those with lowly expressed GCLC. Hence, GCLC showed overexpression in LUAD and was correlated with patient prognosis.

### Identification of GCLC-relevant genes through integrating differential expression analysis and WGCNA

We further explored potential molecular mechanisms underlying GCLC. In accordance with the threshold of adjusted *p*<0.05, 24,620 genes presented the aberrant expression in LUAD versus controls (Fig. 1D; Supplementary table 2). In addition, 8,682 genes were differently expressed between lowly and highly expressed GCLC specimens (Fig. 1E; Supplementary table 3). Among them, 2,828 genes exhibited the significant up-regulation as well as 2,284 presented the down-regulation both in LUAD versus controls and high versus low GCLC expression LUAD (Fig. 1F; Supplementary figure 1A, B; Supplementary table 4), namely GCLC-related differentially expressed genes (DEGs).

**Fig. 2:**
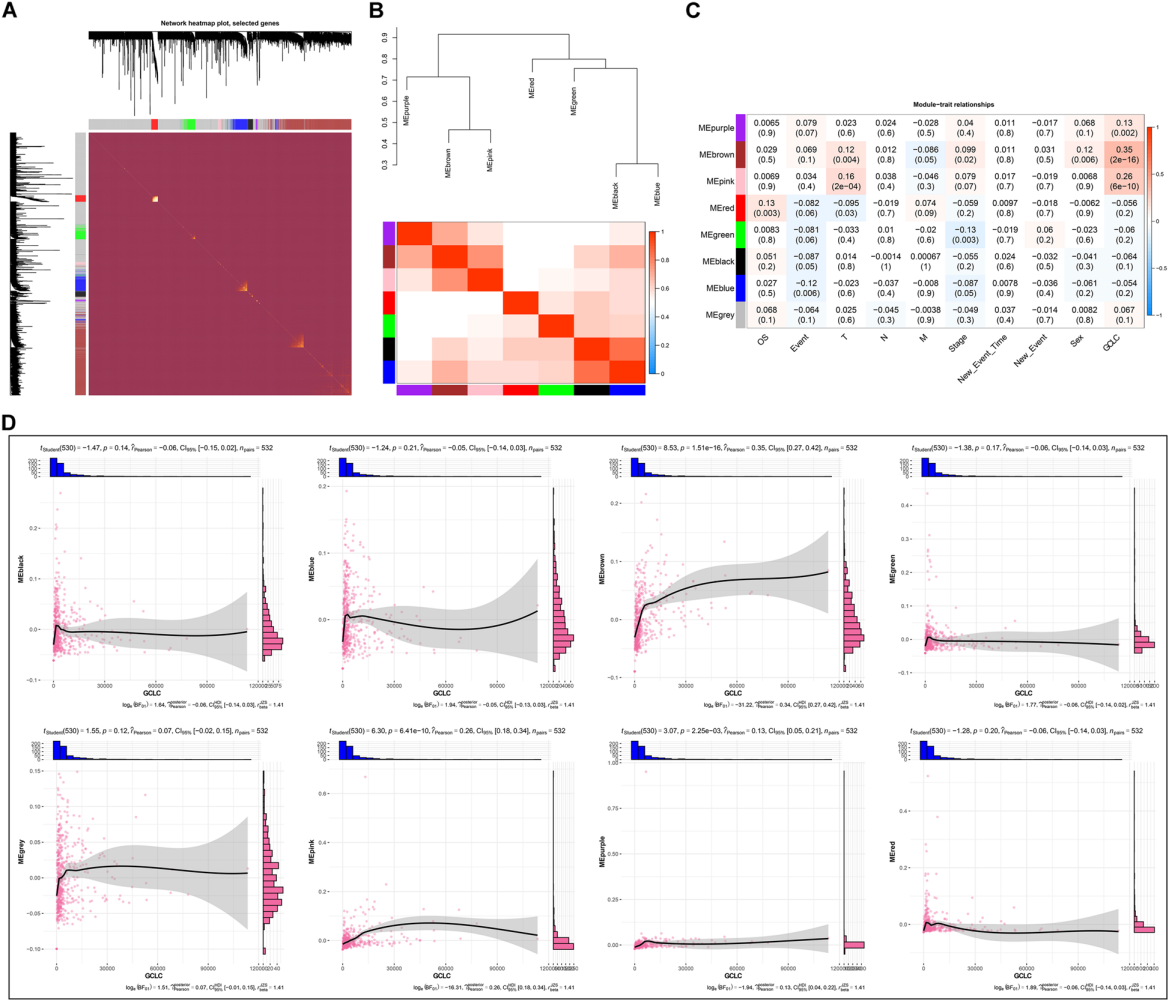
Establishment co-expression modules based upon GCLC-related DEGs. **A** Network heatmap plot in accordance with GCLC-related DEGs across LUAD samples (*n=*532). High co-expression interconnectedness is displayed by gradually saturated yellow and red. **B** Module eigengene dendrogram (upper) and eigengene network heatmap (below). In the heatmap, blue exhibits low adjacency, and red denotes high adjacency. **C** The relationships of module eigengenes with clinical traits and GCLC. Red denotes positive interaction, with blue denoting negative interaction. **D** Scatter plots displaying the relationships of the MEs of modules with GCLC. Pearson’s test was used for correlation analysis

A network correlation analysis was implemented in TCGA-LUAD samples on the basis of GCLC-related DEGs utilizing WGCNA (Supplementary figure 2A). Considering both scale independence and mean connectivity, soft thresholding power was set as 13, satisfying a scale-free network (Supplementary figure 2B). Highly interconnected genes were merged into 8 modules (Supplementary figure 2C, D; Fig. 2A). In addition, we summed up the module-module interactions through hierarchical clustering dendrograms of their eigengenes as well as an eigengene network (Fig. 2B). To comprehend the significance of the modules, this work conducted a correlation analysis on the identified module eigengenes (MEs) with clinical characteristics and GCLC (Fig. 2C). We found that the MEs of blue module were positively connected to event; those of red module presented the positive interactions with T; those of brown module had the positive connections to T, M, stage, sex, and GCLC; those of pink module were positively associated with GCLC. Above findings were further demonstrated by the high connection of gene significance (GS) with module membership (MM) (Supplementary figure 3A-H). In accordance with the strongest interaction of MM in brown module with GS for GCLC, genes in the module appeared to present high connections to GCLC (Supplementary table 5), which were considered as GCLC-relevant genes. Among the merged modules, the MEs of brown module exhibited the strongest connection to GCLC, further proving the high association of brown module genes with GCLC (Fig. 2D). Altogether, our findings identified potential GCLC-relevant genes in the context of LUAD.

### GCLC-relevant genes are associated with cell cycle and DNA replication in LUAD

**Fig. 3:**
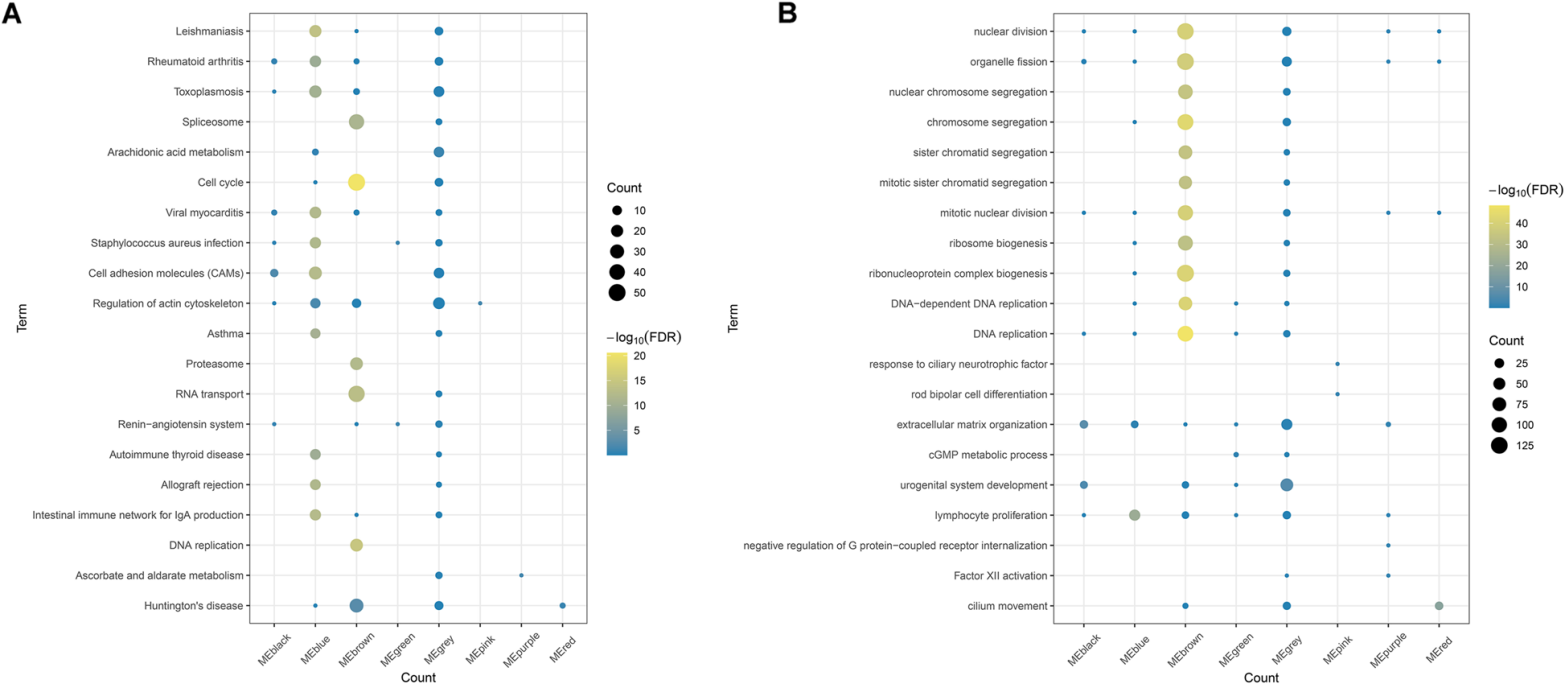
Functional enrichment analysis of module genes. **A** KEGG pathways enriched by module genes. **B** GO enrichment results of module genes

Next, we assessed biological functions and involved pathways of the genes in each module. As a result, brown module genes were found to be in relation to cell cycle (Fig. 3A), and DNA replication (Fig. 3B), indicating the involvement of GCLC-relevant genes in LUAD progression.

### Establishment of a GCLC-based model for LUAD prognosis prediction

**Fig. 4:**
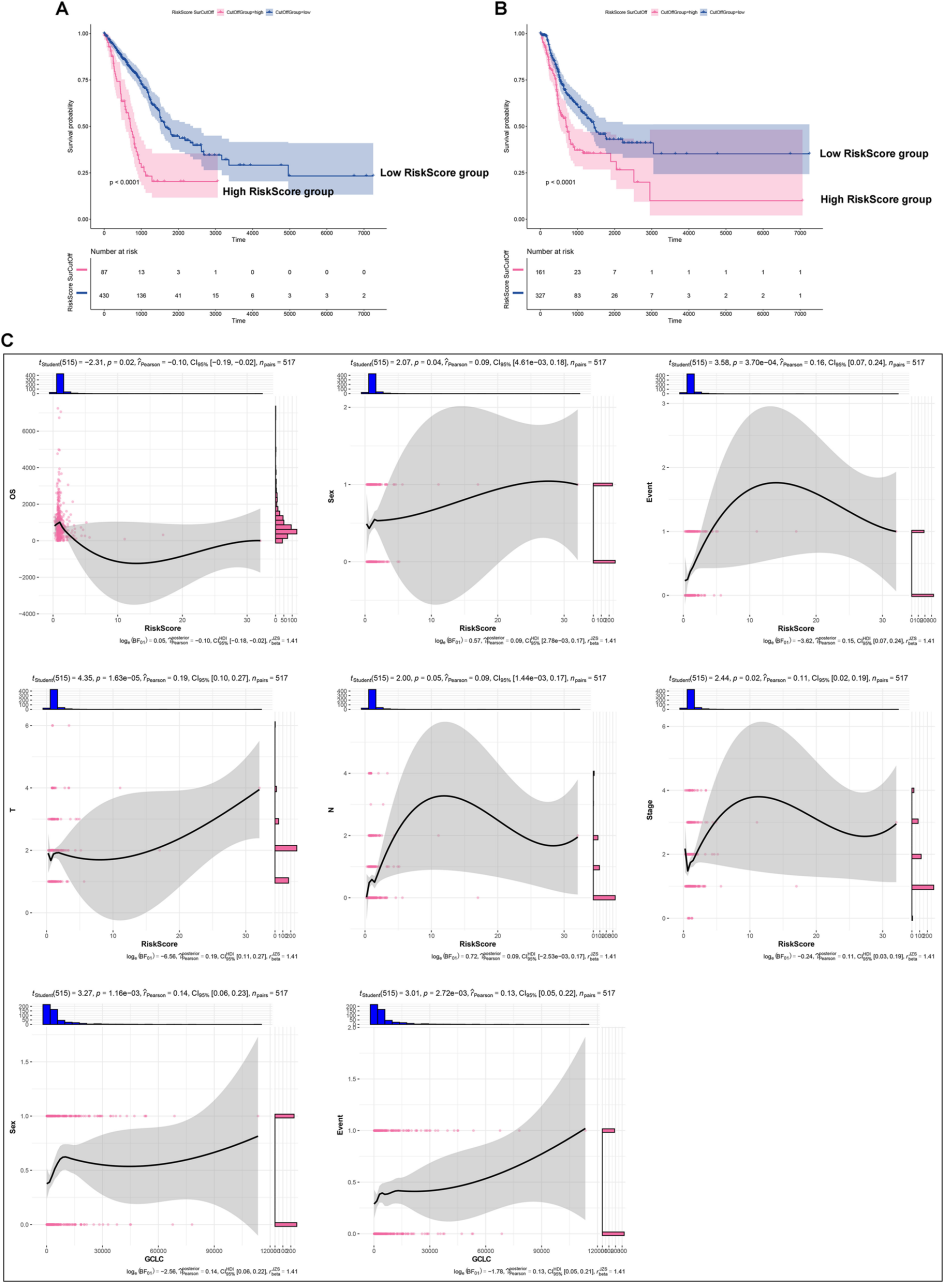
Generation of a GCLC-based model for survival prediction. **A**, **B** Survival probability of low- (*n=*430) and high- (*n=*87) RiskScore patients in the discovery set. **B** Survival probability of low- (*n=*327) and high- (*n=*161) RiskScore patients in the test set. Survival difference was evaluated via log rank test. **C** Correlations of RiskScore and GCLC with clinical parameters across LUAD patients (*n=*517). Pearson’s test was used for correlation analysis

In Supplementary figure 4A, 25 GCLC-relevant genes with *p*<0.05 (GAPDH, E2F7, UCK2, ANLN, HMMR, ECT2, FKBP4, FAM207A, PKM, ARHGAP11A, H2AFX, CCNB1, PRC1, DLGAP5, HMGA1, CDC25C, KPNA2, RRM2, HJURP, MESD, CDKN3, PBK, PLK1, KNL1, and CENPH) exhibited the significant connections with LUAD survival, indicating their possibility as prognostic biomarkers of LUAD. These genes were further employed for the generation of a multivariate-cox regression model composed of CDC25C, CENPH, E2F7, and KNL1, with the formula of RiskScore = 0.001 * CDC25C expression + (-0.001) * CENPH expression + 0.001 * E2F7 expression + (-0.001) * KNL1 expression (Supplementary figure 4B, C). Both in the discovery and test sets, high-RiskScore group presented the poorer overall survival (OS) relative to low-RiskScore group (Fig. 4A, B). This was indicative of the well performance of RiskScore in prognostication. Additionally, RiskScore was negatively linked with OS as well as positively correlated to sex, event, T, N, and stage (Fig. 4C). Meanwhile, GCLC displayed the positive connections to sex and event. Therefore, the GCLC-based model enabled to accurately predict LUAD prognosis and was closely linked with clinical characteristics.

### The GCLC-based model acts as an independent prognostic factor of LUAD

**Fig. 5:**
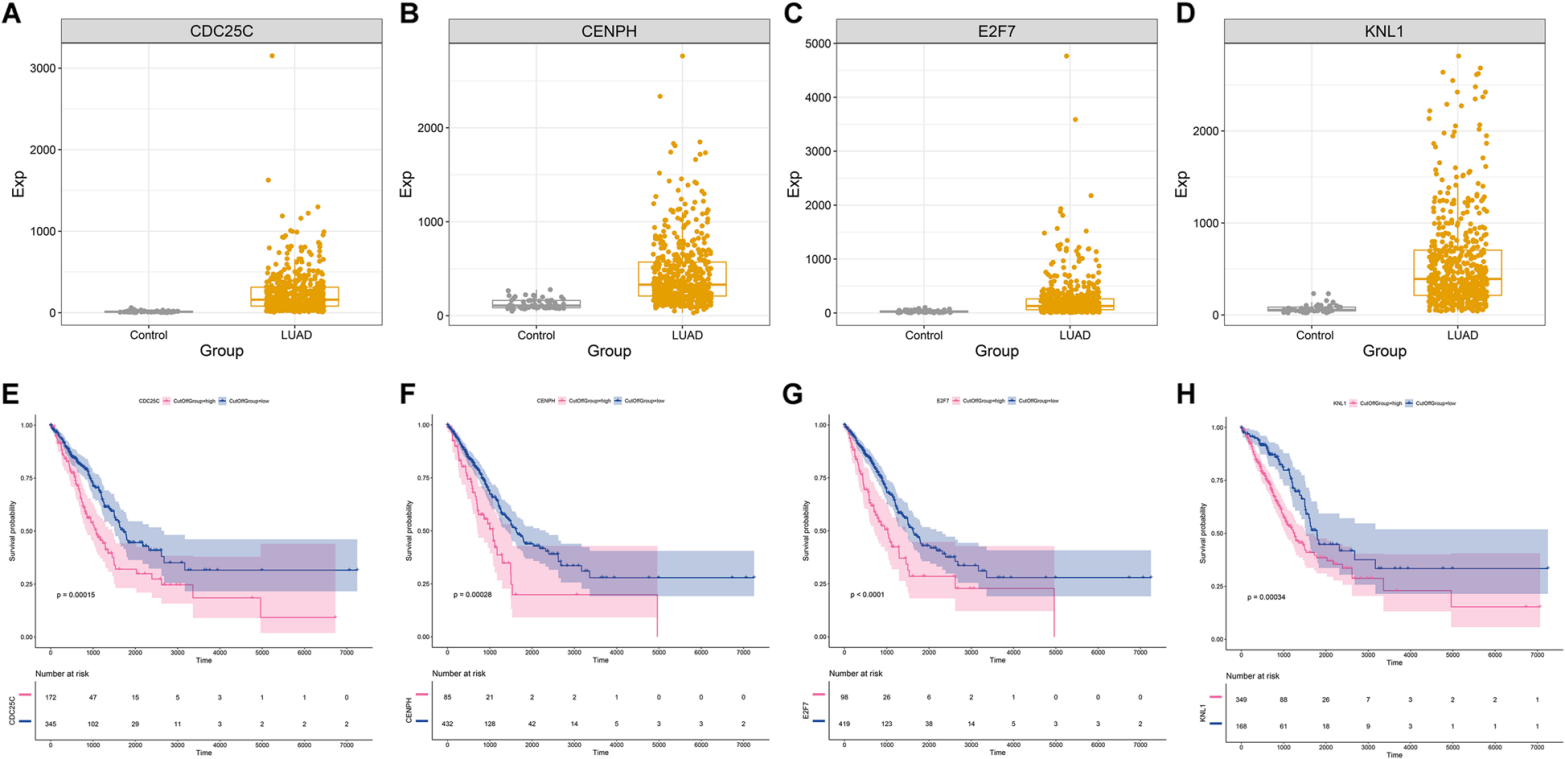
Assessment of the prognostic significance of the genes in the GCLC-based model. **A**-**D** Differential expression of (**A**) CDC25C, **B** CENPH, **C** E2F7, and **D** KNL1 in LUAD (*n=*525) and control (*n=*60) tissue specimens. Student’s test was adopted for comparing between groups. **E**-**H** Survival analyses of LUAD patients with lowly versus highly expressed (**E**) CDC25C (345 versus 172), **F** CENPH (432 versus 85), **G** E2F7 (419 versus 98), and **H** KNL1 (168 versus 349). Survival difference was assessed via log rank test

The four genes from the multivariate-cox regression model: CDC25C, CENPH, E2F7, and KNL1 exhibited the remarkable up-regulation in LUAD relative to controls (Fig. 5A-D). In addition, their up-regulation was significantly in relation to worse OS outcomes (Fig. 5E-H). Considering the uni- and multivariate-cox regression results, RiskScore acted as an independent prognostic factor (Supplementary figure 5A, B). Next, to facilitate clinical practice, a nomogram was generated, composed of RiskScore and common clinical characteristics (T, N, stage, and new event) (Supplementary figure 5C), which owned the well efficacy in prediction of 1-, 3- and 5-year survival (Supplementary figure 5D). Altogether, the GCLC-based model served as an independent prognostic factor of LUAD, and the nomogram could be considered for clinical use.

### GCLC is closely linked with antitumor immunity and immunotherapy response of LUAD

**Fig. 6:**
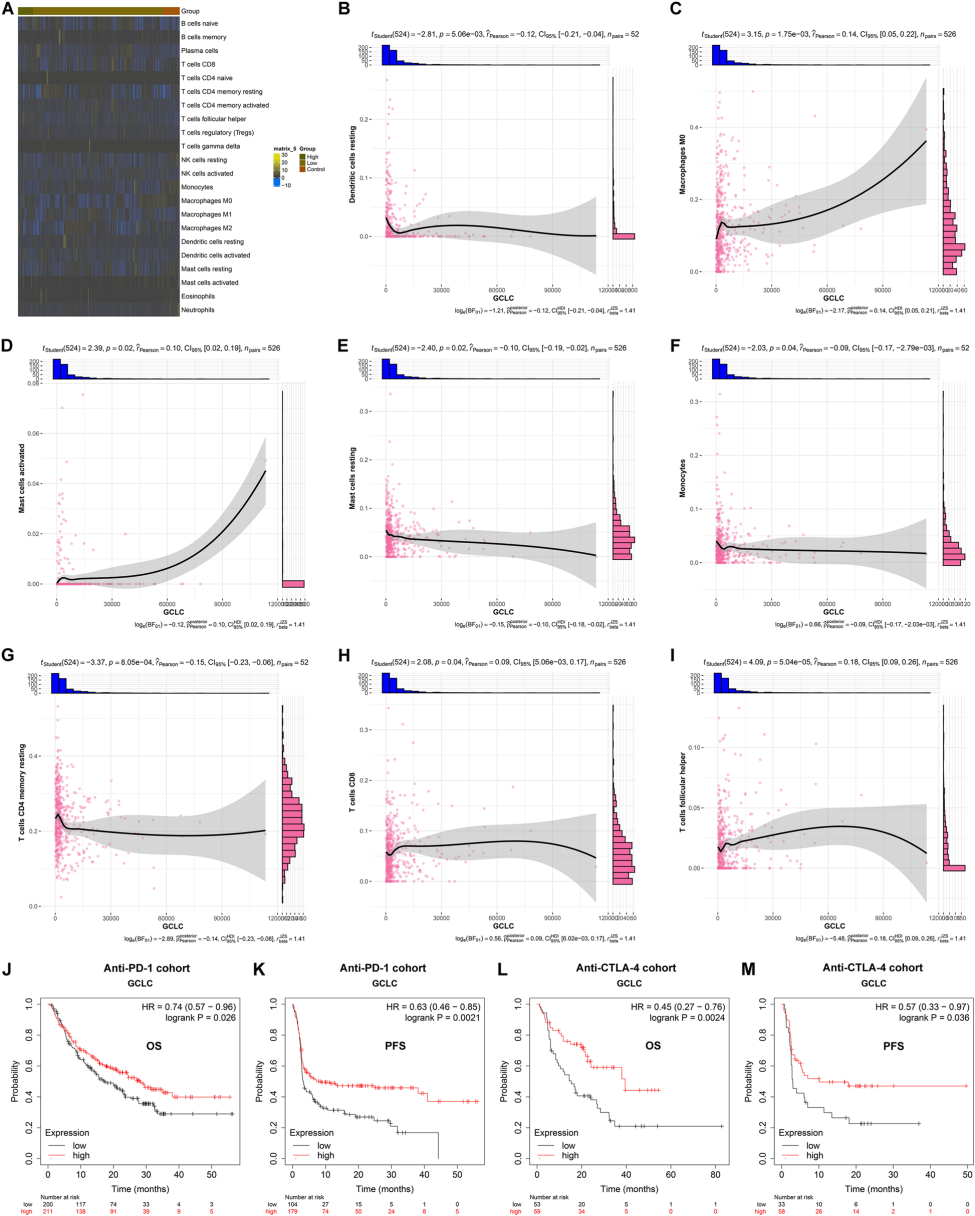
Relationships of GCLC with antitumor immunity. **A** The fraction of each immune composition across control (*n=*59), low (*n=*471) and high (*n=*55) GCLC expression LUAD tissues. **B**-**I** Associations of GCLC with (**B**) dendritic cells resting, **C** macrophages M0, **D** mast cells activated, **E** mast cells resting, **F** monocytes, **G** T cells memory resting, **H** T cells CD8, and **I** T cells follicular helper. **J**, **K** Comparison of overall survival (OS; *n=*411) and progression-free survival (PFS; *n=*283) between solid tumor patients with high GCLC expression and those with low GCLC expression in anti-PD-1 treatment cohort. Pearson’s test was utilized for correlation analysis. **L**, **M** Comparison of OS (*n=*112) and PFS (*n=*91) between solid tumor patients with high GCLC expression and those with low GCLC expression in anti-CTLA-4 treatment cohort. Survival difference was assessed via log rank test

To investigate the role of GCLC in antitumor immunity of LUAD, different immune compositions were firstly investigated in LUAD with lowly and highly expressed GCLC (Fig. 6A). Especially, GCLC presented the negative connections with resting dendritic cells, resting mast cells, monocytes, resting memory T cells, but displayed the positive associations with M0 macrophages, activated mast cells, CD8+ T cells, and follicular helper T cells (Fig. 6B-I). In addition, GCLC-based RiskScore and most GCLC-relevant prognostic genes displayed the positive connections with activated memory CD4+ T cells, follicular helper T cells, gamma delta T cell, and M0 macrophages, with the negative associations with other immune compositions (Supplementary figure 6A). This demonstrated that GCLC might participate in modulation of immune cell infiltration in tumors. It was also found that GCLC, RiskScore and relevant prognostic genes were positively interacted with most immune checkpoint molecules (Supplementary figure 6B). To further investigate the associations between GCLC and immunotherapeutic response, two immunotherapy cohorts (anti-PD-1 and anti-CTLA-4) were included via Kaplan-Meier Plotter. The results showed that solid tumor patients with high GCLC expression had better overall survival (OS) and progression-free survival (PFS) outcomes compared with those with low GCLC expression after anti-PD-1 or anti-CTLA-4 treatment (Fig. 6J-M), indicating that patients with high GCLC expression might benefit from anti-PD-1 or anti-CTLA-4 treatment. Therefore, GCLC was closely associated with antitumor immunity and immunotherapy response of LUAD.

### GCLC and relevant prognostic genes are affected by DNA methylation modifications and genetic mutations

**Fig. 7:**
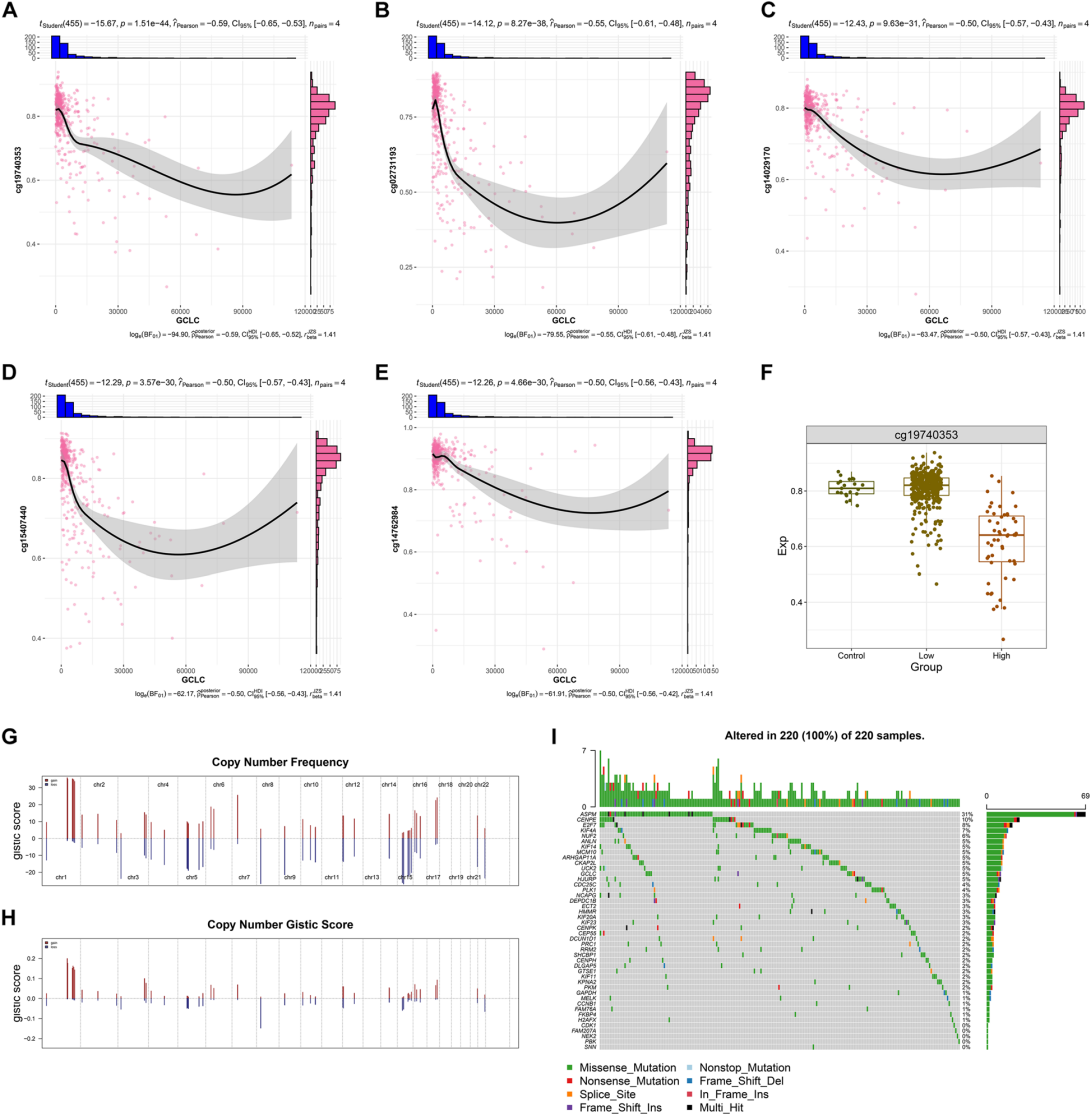
DNA methylation modification and genetic variations of GCLC and relevant prognostic genes. **A**-**E** Relationships of GCLC expression with beta values of (**A**) cg19740353, **B** cg02731193, **C** cg14029170, **D** cg15407440, and **E** cg14762984 across LUAD samples (*n=*455). Pearson’s test was utilized for correlation analysis. **F** Difference in beta values of cg19740353 in control (*n=*20), lowly (*n=*401) and highly (*n=*51) expressed GCLC LUAD tissues. **G**, **H** Copy number frequency and GISTIC score of GCLC and relevant prognostic genes. **I** Waterfall plot illustrating the mutated frequency of GCLC and relevant prognostic genes across LUAD samples (*n=*220)

DNA methylation of GCLC and relevant prognostic genes was then evaluated in LUAD. Supplementary figure 7 illustrated the connections of their expression with beta values of CpGs. Notably, GCLC expression was strongly and negatively linked with beta values of cg19740353, cg02731193, cg14029170, cg15407440, and cg14762984 (Fig. 7A-E). Among them, cg19740353 exhibited the remarkable difference in beta value between controls, lowly and highly expressed GCLC LUAD (Fig. 7F). In comparison to controls or low GCLC LUAD, beta value of cg19740353 was lower in highly expressed GCLC LUAD. This indicated that up-regulation of GCLC was potentially modulated by hypomethylation in cg19740353. In addition, copy number amplifications and deletions frequently occurred in GCLC and relevant prognostic genes (Fig. 7G, H). Analysis of somatic mutation unveiled that ASPM exhibited the most frequent mutation (31%), followed by CENPE (10%), E2F7 (8%), KIF4A (7%), NUF2 (6%), etc. in LUAD (Supplementary figure 8; Fig. 7I). Collectively, GCLC and relevant prognostic genes were potentially affected by DNA methylation mechanisms and genetic mutations.

### GCLC and relevant prognostic genes are post-transcriptionally regulated by non-coding RNAs

**Fig. 8:**
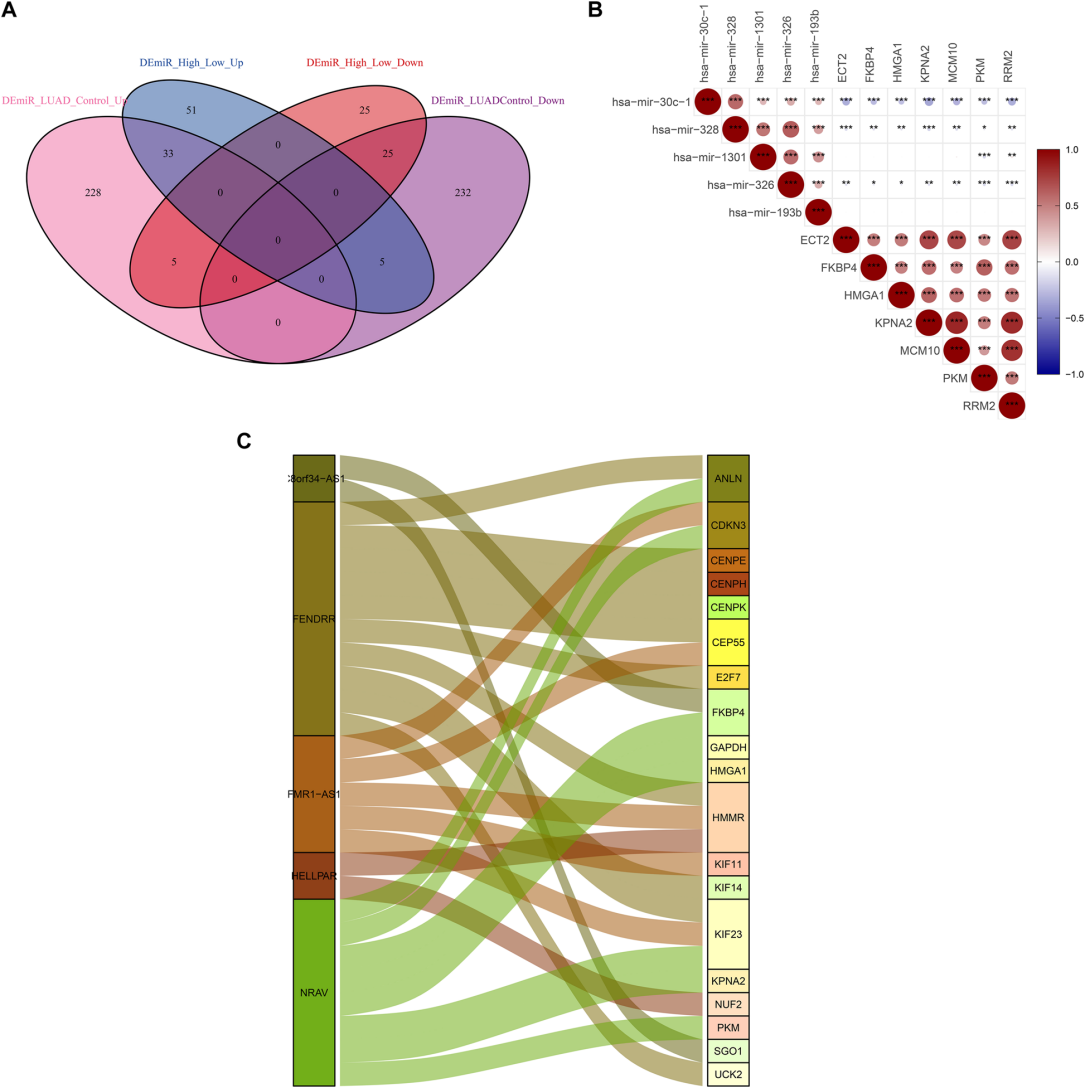
Potential post-transcriptional regulation of GCLC and relevant prognostic genes. **A** The shared miRNAs between above two miRNA sets. **B** Relationships of the shared miRNAs with GCLC and relevant prognostic genes. Pearson’s test was employed for correlation analysis. **C** Sankey diagram visualizing the interactions of lncRNAs with GCLC and relevant prognostic genes. *p<0.05; **p<0.01; ***p<0.001

With adjusted *p*<0.05, 528 miRNAs were found to be aberrantly expressed in LUAD relative to control tissues (Supplementary figure 9A; Supplementary table 6). In addition, 144 miRNAs with different expression were determined between lowly and highly expressed GCLC LUAD (Supplementary figure 9B; Supplementary table 7). Furthermore, 33 miRNAs presented the up-regulation both in LUAD versus controls and lowly versus highly expressed GCLC, with 25 presenting the down-regulation (Fig. 8A; Supplementary figure 9C; Supplementary table 8). Among them, hsa-mir-30c-1, hsa-mir-328, hsa-mir-1301, hsa-mir-326, and hsa-mir-193b were negatively connected to ECT2, FKBP4, HMGA1, KPNA2, MCM10, PKM, and RRM2 (Fig. 8B). In addition, several lncRNAs including C8orf34-AS1, FENDRR, FMR1-AS1, HELLPAR, and NRAV presented the notable interactions with GCLC and relevant prognostic genes (Fig. 8C). Above data revealed the potential post-transcriptional regulation of GCLC and relevant genes by non-coding RNAs.

### Transcriptional, m^6^A and SUMOylation modification of GCLC and relevant prognostic genes

**Fig. 9:**
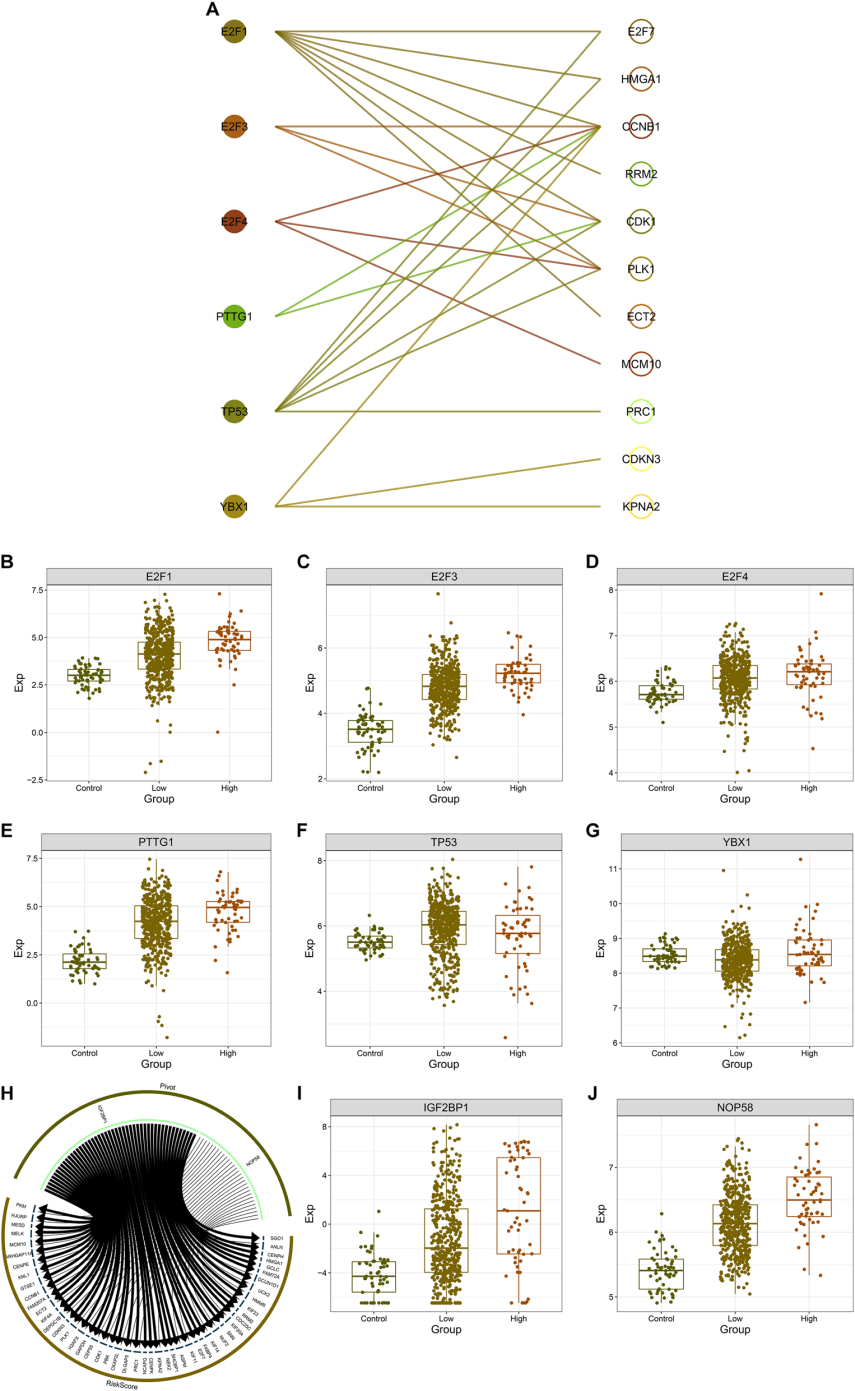
Potential regulation of GCLC and relevant prognostic genes by transcription factors, m^6^A and SUMOylation modification in LUAD. **A** Associations between transcription factors and GCLC and relevant prognostic genes. **B**-**G** The transcript levels of (**B**) E2F1, **C** E2F3, **D** E2F4, **E** PTTG1, **F** TP53, and **G** YBX1 across controls (*n=*59), low (*n=*471) and high (*n=*55) GCLC expression LUAD tissues. **H** Relationships of IGF2BP1, and NOP58 with GCLC and relevant prognostic genes. **I**, **J** The transcript levels of (**I**) IGF2BP1, and (**J**) NOP58 across controls (*n=*59), lowly (*n=*471) and highly (*n=*55) expressed GCLC LUAD

Next, it was found that E2F1, E2F3, E2F4, PTTG1, TP53, and YBX1 transcription factors displayed the remarkable interactions with GCLC-relevant prognostic genes (comprising E2F7, HMGA1, CCNB1, RRM2, CDK1, PLK1, ECT2, MCM10, PRC1, CDKN3, and KPNA2) (Fig. 9A). These transcription factors were remarkably overexpressed in LUAD with GCLC up-regulation in comparison to controls or LUAD with GCLC down-regulation (Fig. 9B-G). IGF2BP1 (a m^6^A regulator), and NOP58 (a SUMOylation regulator) were closely connected with GCLC and relevant prognostic genes (Fig. 9H). Both presented the remarkable up-regulation in highly expressed GCLC LUAD relative to controls or lowly expressed GCLC LUAD (Fig. 9I, J). These data uncovered the possible transcriptional, m^6^A and SUMOylation modification mechanisms of GCLC and relevant prognostic genes in the context of LUAD.

## Discussion

Ferroptosis has been evidenced to be connected with LUAD, and GCLC participates in the key process of ferroptosis [22]. In this work, GCLC displayed the overexpression in LUAD, and was correlated to worse survival, consistent with a prior study [13]. The GCLC-based model for accurate prognostication was built for the first time, composed of CDC25C, CENPH, E2F7, and KNL1. For the facilitation of clinical application, the nomogram was also proposed based on the GCLC-based model. Nonetheless, the model requires further verification in future prospective cohorts.

LUAD is an extremely heterogeneous malignancy that involves complex crosstalk of malignant cells with tumor microenvironment [23, 24]. Immune cells dominate the tumor microenvironment, which nearly affect each stage of tumorigenesis via direct interactions with malignant cells [25]. GCLC was found to be significantly in relation to the infiltrating immune cells, notably resting dendritic cells, resting mast cells, monocytes, resting memory T cells, M0 macrophages, activated mast cells, CD8+ T cells, and follicular helper T cells [24]. Combining the connections of GCLC with immune checkpoints, it was inferred the role of GCLC in antitumor immunity regulation during LUAD. Similarly, Zhang et al. determined crucial ferroptosis regulators in LUAD, and proposed that RRM2 facilitated immune infiltration through ferroptosis inhibition [26]. In two anti-PD-1 and anti-CTLA-4 immunotherapy cohorts, solid tumor patients with high GCLC expression had better prognostic outcomes in comparison to those with low GCLC expression, revealing that high GCLC expression was in relation to better immunotherapeutic response.

Epigenetic alterations denote as genetic modifications that modulate gene expression without any change in the underlying nucleotide sequence [27]. DNA methylation is the selective addition of methyl groups to the CpG site to take shape 5-methylcytosine, which has been extensively proven to be involved in LUAD [28]. For instance, a previous study reported that hypermethylation status of ALDH2 in LUAD that is linked with stem cell-related pathways [29]. The possible methylation sites of GCLC and relevant prognostic genes were predicted in our work, and cg19740353, cg02731193, cg14029170, cg15407440, and cg14762984 were found for GCLC. Cancer genomics offers broad insights into cancer-associated genes [30]. The study investigated that, genetic alterations of GCLC and relevant prognostic genes frequently occurred in LUAD, especially ASPM mutation [31].

Non-coding RNAs exert essential roles in post-transcriptional regulation [32, 33]. This work determined aberrantly expressed miRNAs (hsa-mir-30c-1, hsa-mir-328, hsa-mir-1301, hsa-mir-326, and hsa-mir-193b) as well as lncRNAs (C8orf34-AS1, FENDRR, FMR1-AS1, HELLPAR, and NRAV) that possibly contributed to the aberrant expression of GCLC and relevant prognostic genes in LUAD via the post-transcriptional modulation mechanisms. Deregulated E2F1, E2F3, E2F4, PTTG1, TP53, and YBX1 transcription factors were closely connected to GCLC and relevant prognostic genes in LUAD, indicating the roles in mediating their transcription. m^6^A modification represents the most abundant modification within RNAs [34]. Evidence has demonstrated that IGF2BP1 triggers the malignant phenotypes of LUAD partly via a m^6^A-dependent manner [35, 36]. Consistently, this study identified that IGF2BP1 was overexpressed in LUAD. In addition, IGF2BP1-mediated m^6^A methylation potentially modulated the expression of GCLC and relevant genes. SUMOylation is a reversible post-translational modification [37–39], and its deregulation extensively participates in tumorigenesis, immune response, cell cycle progression, etc. [40]. In addition, a remarkable function of SUMOylation in molecular pathways is to govern the cellular deaths [41]. NOP58 has been proven to associate with LUAD recurrence [42]. In the present study, NOP58 was found to present the higher transcript level in LUAD with GCLC up-regulation in comparison to normal tissues or LUAD with GCLC down-regulation. Thus, NOP58 was positively correlated to GCLC expression in LUAD, indicating that NOP58 might potentially modulate the post-translational modification of GCLC and relevant genes.

However, several limitations of this study should be pointed out. Firstly, the analysis mainly relies on a constrained number of LUAD samples from public datasets, and more external validation datasets are required for validating the clinical implications of GCLC and relevant prognostic genes in LUAD prognosis and antitumor immunity. In the future, large-scale verification with more complete clinical characteristics will thus be crucial. Secondly, although this work unveiled the potential multi-omics regulatory mechanisms of GCLC and its relevant prognostic genes, further multi-omics analytical methods may also better aid our efforts to understand the multi-omics regulatory mechanisms. Furthermore, in-depth experiments will be performed to prove our conclusions in our future research.

## Conclusion

In summary, this work demonstrates the clinical implications of GCLC and relevant genes for prognosis and antitumor immunity of LUAD. Additionally, we uncover the complex multi-omics mechanisms of GCLC and relevant genes in LUAD. Altogether, our findings provide substantial evidence to support the clinical potential of GCLC and relevant genes as prognostic biomarkers of LUAD. In our future research, we will explore the possibility of GCLC and relevant genes as therapeutic targets for LUAD. Moreover, regulatory molecular mechanisms underlying GCLC and relevant genes in LUAD will be investigated in depth.

### Supplementary Information

Supplementary Material 1.

Supplementary Material 2.

## Data Availability

The datasets analyzed during the current study are available in the TCGA repository, [https://portal.gdc.cancer.gov].

## Abbreviations

LUAD: Lung adenocarcinoma
GCLC: Glutamate-cysteine ligase catalytic subunit
TCGA: The Cancer Genome Atlas
WGCNA: Weighted correlation network analysis
ME: Module eigengene
MM: Module membership
GS: Gene significance
KEGG: Kyoto Encyclopedia of Genes and Genomes
GO: Gene Ontology
miRNA: MicroRNA
lncRNAs: Long non-coding RNAs
m^6^A: N^6^-methyladenosine
OS: Overall survival
