# Supplementary material for: Implications of GCLC in prognosis and immunity of lung adenocarcinoma and multi-omics regulation mechanisms
Source: BMC Pulm Med. 2024 May 15;24:239. doi: 10.1186/s12890-024-03052-3 (PMC11095029; doi:10.1186/s12890-024-03052-3)
Supplement: Supplementary file 1 — Supplementary Material 1. [file 12890_2024_3052_MOESM1_ESM.zip › Supplementary figure 3.pdf]

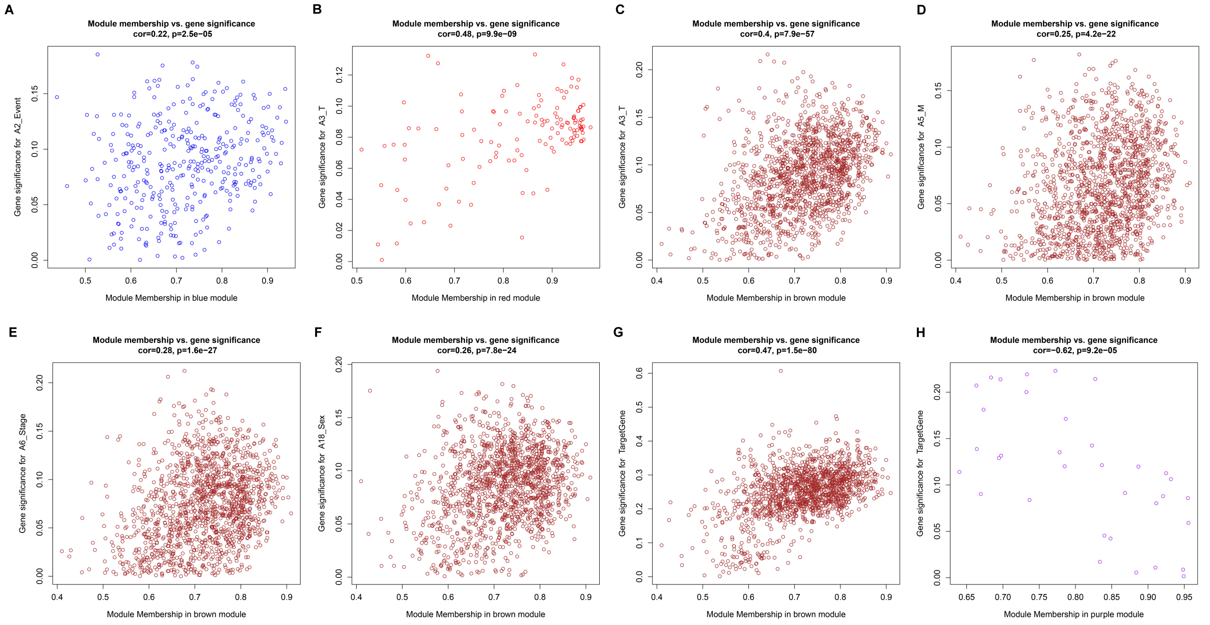

A
Module membership vs. gene significance
cor=0.22, p=2.5e−05
Gene significance for A2_Event
Module Membership in blue module
B
Module membership vs. gene significance
cor=0.48, p=9.9e−09
Gene significance for A3_T
Module Membership in red module
C
Module membership vs. gene significance
cor=0.4, p=7.9e−57
Gene significance for A3_T
Module Membership in brown module
D
Module membership vs. gene significance
cor=0.25, p=4.2e−22
Gene significance for A5_M
Module Membership in brown module
E
Module membership vs. gene significance
cor=0.28, p=1.6e−27
Gene significance for A6_Stage
Module Membership in brown module
F
Module membership vs. gene significance
cor=0.26, p=7.8e−24
Gene significance for A18_Sex
Module Membership in brown module
G
Module membership vs. gene significance
cor=0.47, p=1.5e−80
Gene significance for TargetGene
Module Membership in brown module
H
Module membership vs. gene significance
cor=−0.62, p=9.2e−05
Gene significance for TargetGene
Module Membership in purple module
