# Supplementary material for: Implications of GCLC in prognosis and immunity of lung adenocarcinoma and multi-omics regulation mechanisms
Source: BMC Pulm Med. 2024 May 15;24:239. doi: 10.1186/s12890-024-03052-3 (PMC11095029; doi:10.1186/s12890-024-03052-3)
Supplement: Supplementary file 1 — Supplementary Material 1. [file 12890_2024_3052_MOESM1_ESM.zip › Supplementary figure 8.pdf]

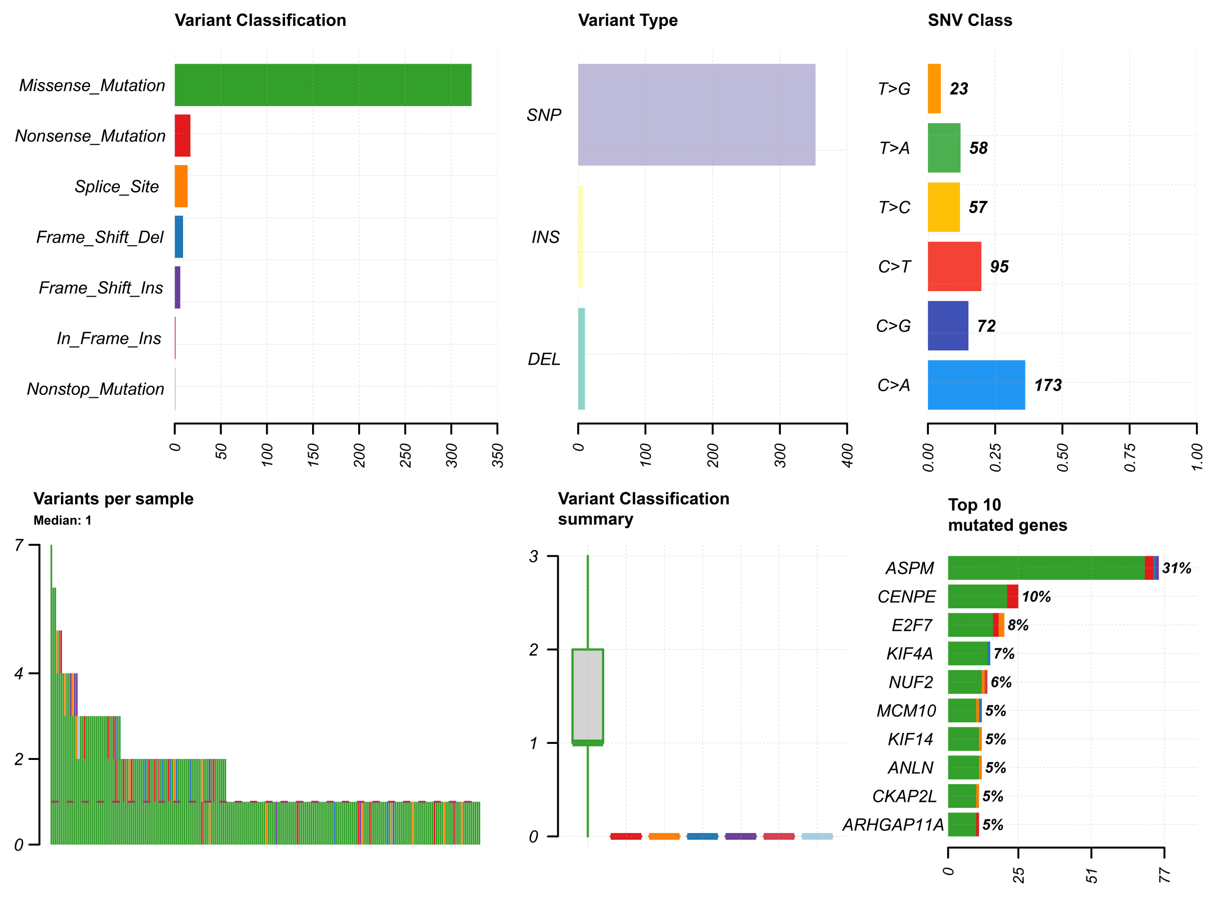
Variant Classification
Missense_Mutation
Nonsense_Mutation
Splice_Site
Frame_Shift_Del
Frame_Shift_Ins
In_Frame_Ins
Nonstop_Mutation
0
50
100
150
200
250
300
350
Variant Type
SNP
INS
DEL
0
100
200
300
400
SNV Class
T>G
23
T>A
58
T>C
57
C>T
95
C>G
72
C>A
173
0.00
0.25
0.50
0.75
1.00
Variants per sample
Median: 1
7
4
2
0
Variant Classification summary
3
2
1
0
Top 10 mutated genes
ASPM
31%
CENPE
10%
E2F7
8%
KIF4A
7%
NUF2
6%
MCM10
5%
KIF14
5%
ANLN
5%
CKAP2L
5%
ARHGAP11A
5%
0
25
51
77
