# Supplementary material for: Implications of GCLC in prognosis and immunity of lung adenocarcinoma and multi-omics regulation mechanisms
Source: BMC Pulm Med. 2024 May 15;24:239. doi: 10.1186/s12890-024-03052-3 (PMC11095029; doi:10.1186/s12890-024-03052-3)
Supplement: Supplementary file 1 — Supplementary Material 1. [file 12890_2024_3052_MOESM1_ESM.zip › Supplementary files.docx]

**Supplementary files:**

Supplementary figure 1. Identification of GCLC-related DEGs. (A) Differential expression of the shared DEGs in LUAD versus controls and high versus low expression of GCLC. (B) Heatmap of the transcript levels of the shared DEGs in controls, lowly and highly expressed GCLC LUAD.

Supplementary figure 2. Establishment co-expression modules based upon GCLC-related DEGs. (A) Sample dendrogram (upper) and heatmap of clinical traits and GCLC (below). (B) Scale independence (left) and mean connectivity (right) under distinct soft threshold powers. (C, D) Gene dendrograms from average linkage hierarchical clustering. The color rows below the dendrogram display the module assignments identified by (C) dynamic tree cut and (D) merged dynamic methods.

Supplementary figure 3. Relationships between co-expression modules and clinical features. (A) A scatter plot of GS for event versus MM in blue module. (B) A scatter plot of GS for T stage versus MM in red module. (C-G) Scatter plots of GS for (C) T, (D) M, (E) stage, (F) sex, and (G) GCLC versus MM in brown module. (H) A scatter plot of GS for GCLC versus MM in purple module.

Supplementary figure 4. Generation of a GCLC-based model in survival prediction. (A, B) Forest diagrams of the uni- and multivariate-cox regression results on GCLC-relevant genes and LUAD survival. (C) The distribution of RiskScore across TCGA-LUAD specimens.

Supplementary figure 5. Construction of a GCLC-based nomogram in prognosis prediction. (A, B) Uni- and multivariate-cox regression results on RiskScore and clinical variables with survival. (C) The nomogram for prognostication. (D) Calibration curves for the nomogram-predicted and actual survival.

Supplementary figure 6. Associations of GCLC-based RiskScore and relevant prognostic genes with immune compositions and immune checkpoint molecules. (A) Bubble diagram illustrating the connections between GCLC-based RiskScore and relevant prognostic genes and the fractions of immune compositions. Blue denotes negative correlation, with yellow denoting positive correlation. (B) The relationships of GCLC-based RiskScore and relevant prognostic genes with the transcript levels of immune checkpoint molecules. Blue represents negative association, with red representing positive association.

Supplementary figure 7. CpG methylation sites corresponding to GCLC and relevant prognostic genes. The size of bubble is proportional to |correlation coefficient|.

Supplementary figure 8. Overview of somatic mutations across LUAD.

Supplementary figure 9. Potential post-transcriptional regulation of GCLC by miRNAs. (A) MiRNAs with aberrant expression in LUAD versus controls. (B) MiRNAs with different expression between lowly and highly expressed GCLC LUAD. (C) The expression of the shared miRNAs across controls, lowly and highly expressed GCLC LUAD.
