# Supplementary figures and images for: Implications of GCLC in prognosis and immunity of lung adenocarcinoma and multi-omics regulation mechanisms
Source: BMC Pulm Med. 2024 May 15;24:239. doi: 10.1186/s12890-024-03052-3 (PMC11095029; doi:10.1186/s12890-024-03052-3)

**A**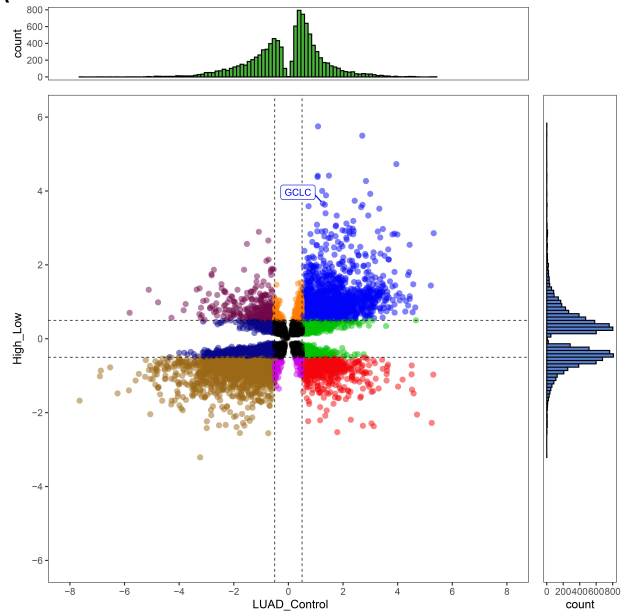**B**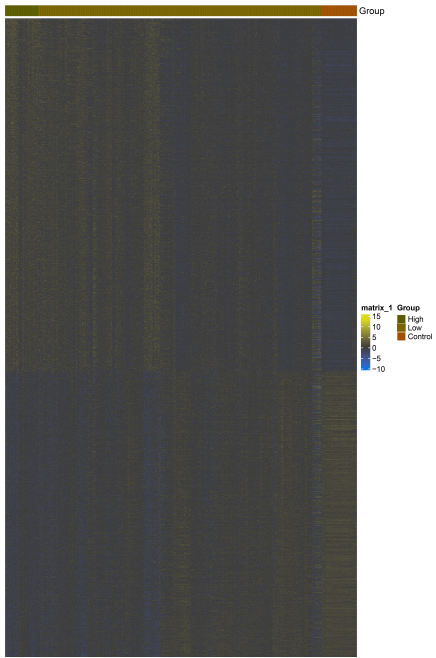

Supplement: Supplementary file 1 — Supplementary Material 1. [file 12890_2024_3052_MOESM1_ESM.zip › Supplementary figure 1.pdf]

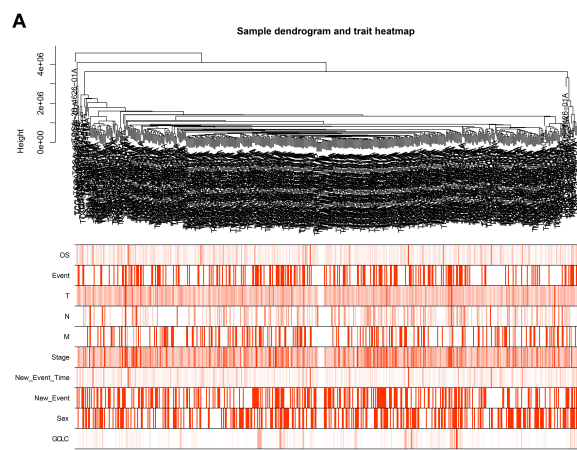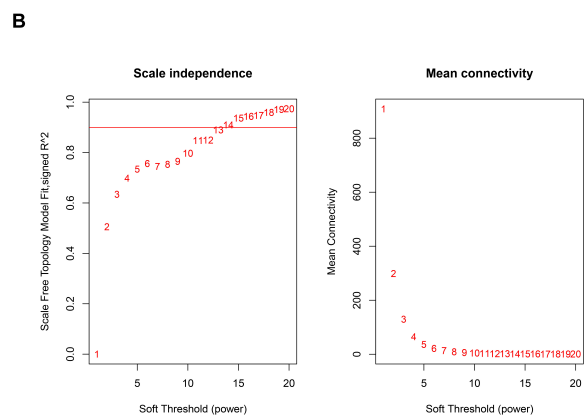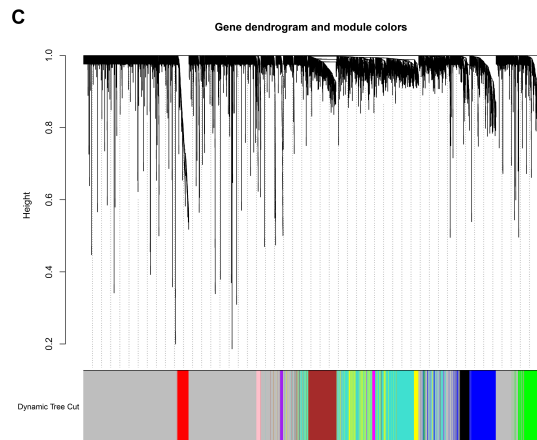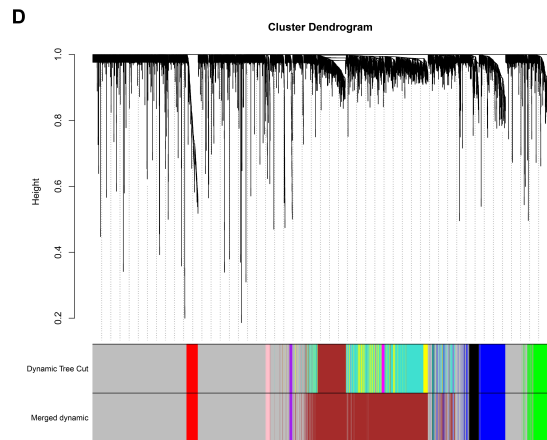

Supplement: Supplementary file 1 — Supplementary Material 1. [file 12890_2024_3052_MOESM1_ESM.zip › Supplementary figure 2.pdf]

**A**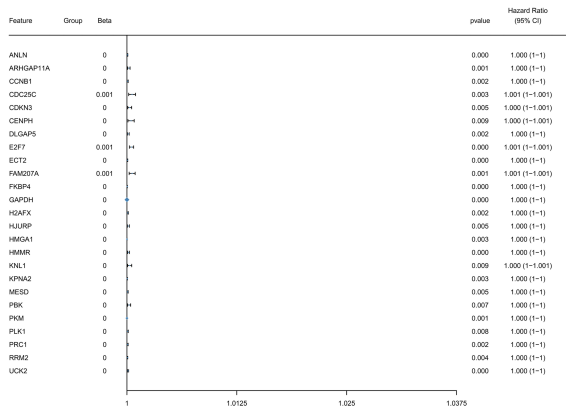**B**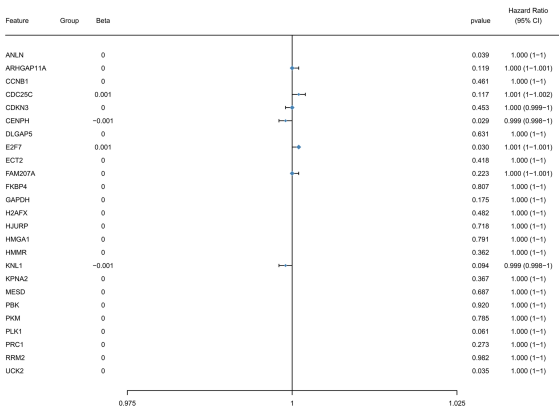**C**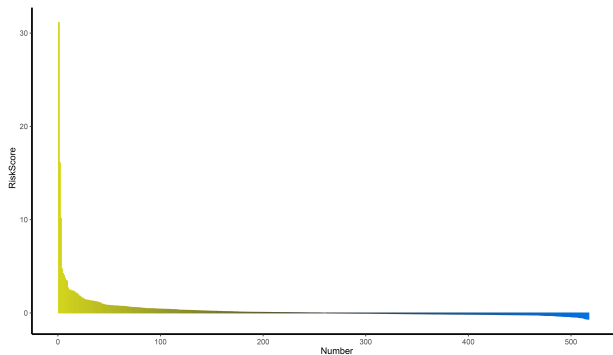

Supplement: Supplementary file 1 — Supplementary Material 1. [file 12890_2024_3052_MOESM1_ESM.zip › Supplementary figure 4.pdf]

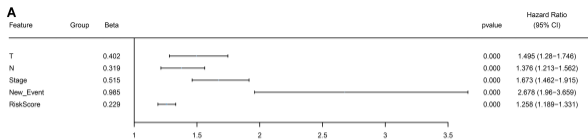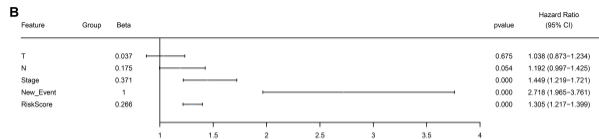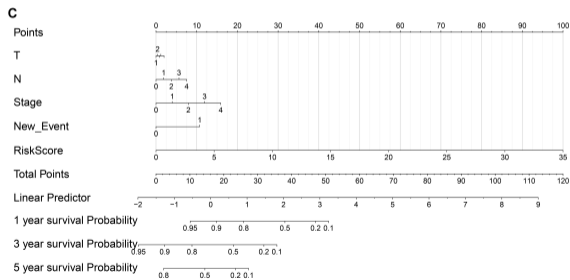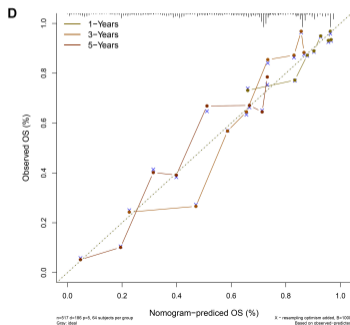

Supplement: Supplementary file 1 — Supplementary Material 1. [file 12890_2024_3052_MOESM1_ESM.zip › Supplementary figure 5.pdf]

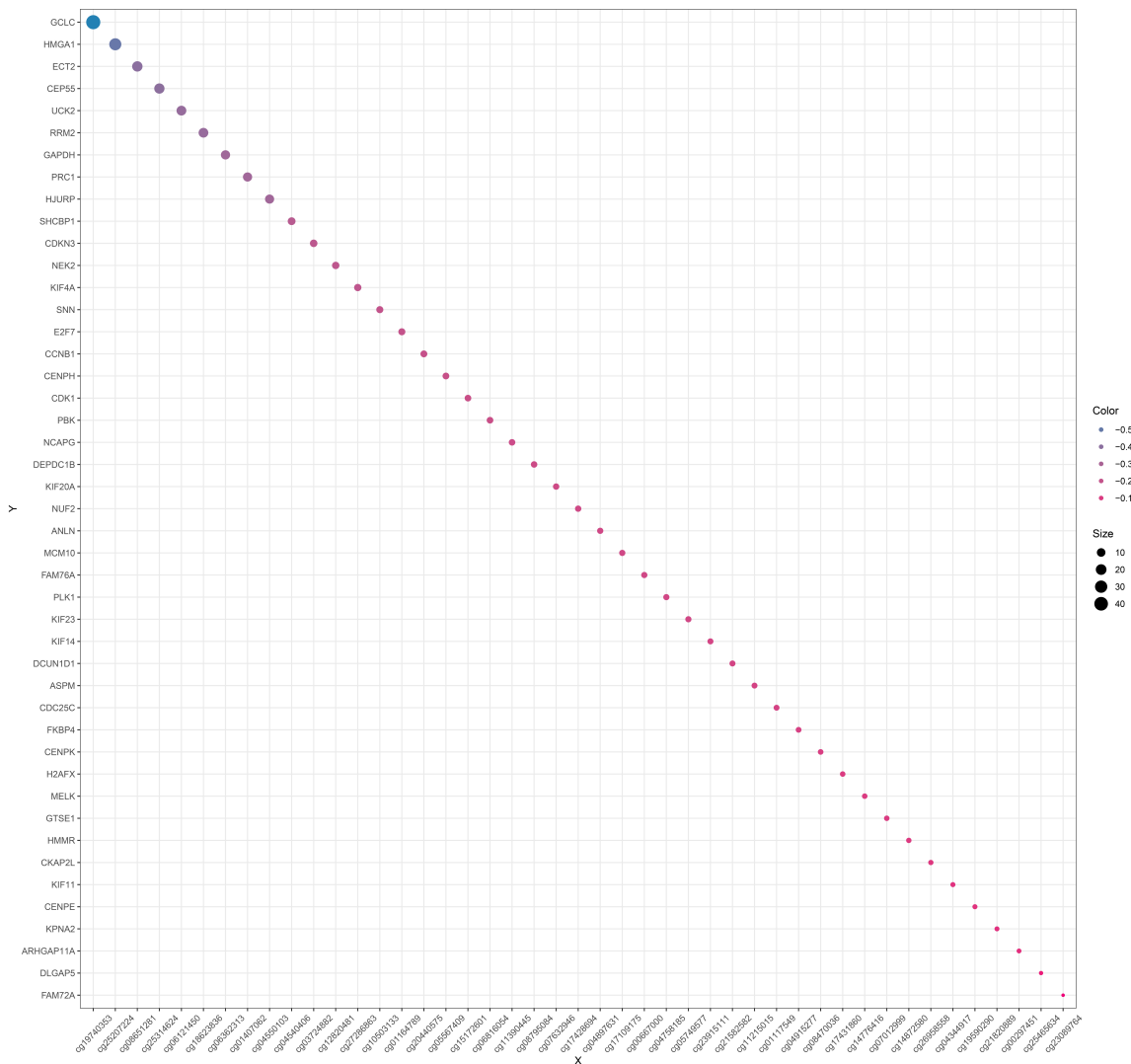

Supplement: Supplementary file 1 — Supplementary Material 1. [file 12890_2024_3052_MOESM1_ESM.zip › Supplementary figure 7.pdf]

**A**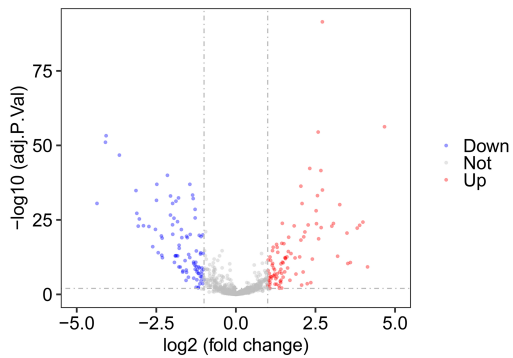**B**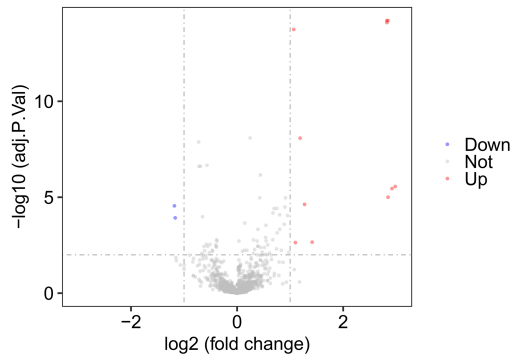**C**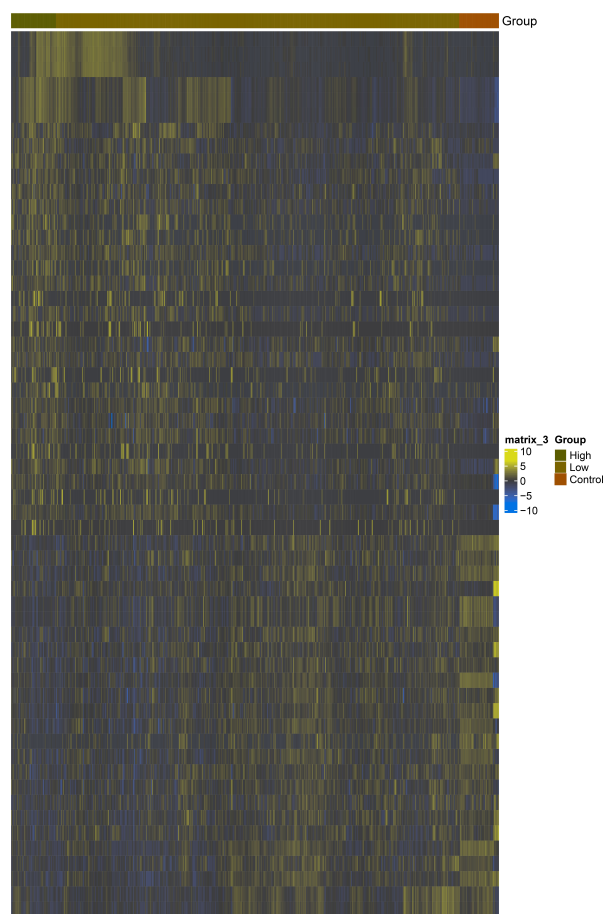

Supplement: Supplementary file 1 — Supplementary Material 1. [file 12890_2024_3052_MOESM1_ESM.zip › Supplementary figure 9.pdf]
