# Supplementary material for: Implications of GCLC in prognosis and immunity of lung adenocarcinoma and multi-omics regulation mechanisms
Source: BMC Pulm Med. 2024 May 15;24:239. doi: 10.1186/s12890-024-03052-3 (PMC11095029; doi:10.1186/s12890-024-03052-3)
Supplement: Supplementary file 1 — Supplementary Material 1. [file Supplementary figure 6.pdf › Supplementary figure 6.pdf]

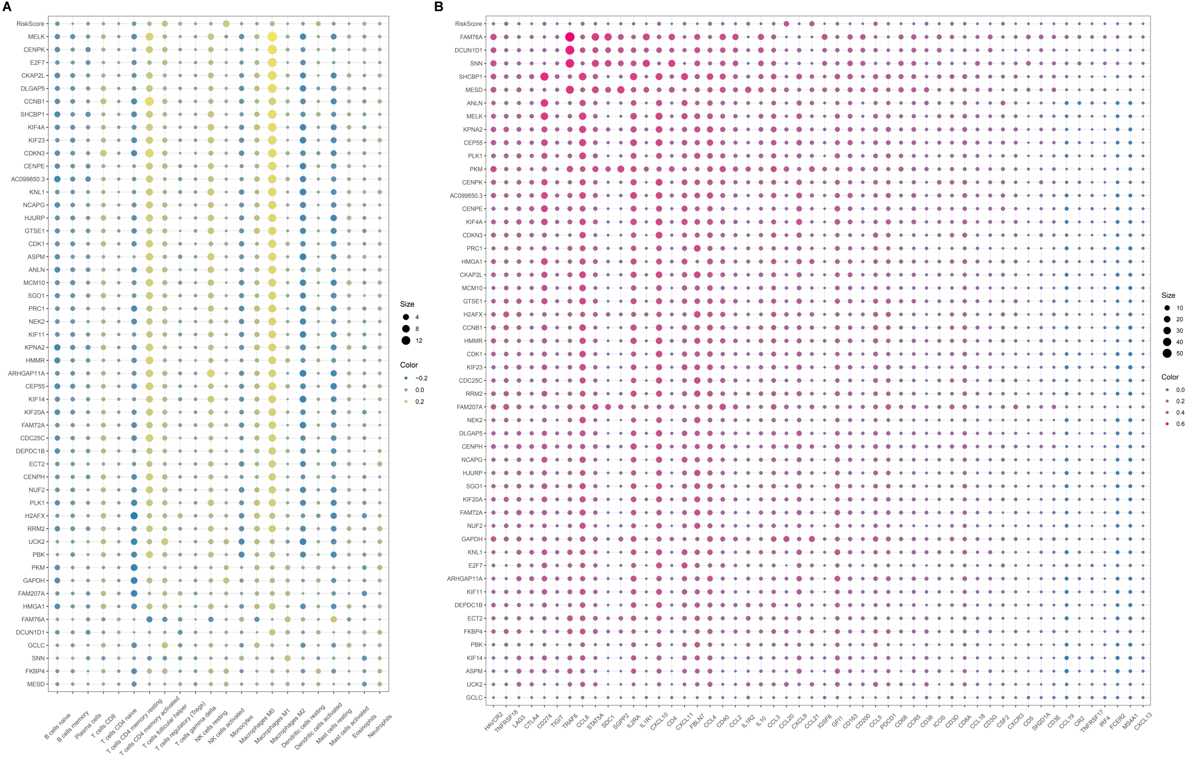
A
RiskScore
MELK
CENPK
E2F7
CKAP2L
DLGAP5
CCNB1
SHCBP1
KIF4A
KIF23
CDKN3
CENPE
AC099850.3
KNL1
NCAPG
HJURP
GTSE1
CDK1
ASPM
ANLN
MCM10
SGO1
PRC1
NEK2
KIF11
KPNA2
HMMR
ARHGAP11A
CEP55
KIF14
KIF20A
FAM72A
CDC25C
DEPDC1B
ECT2
CENPH
NUF2
PLK1
H2AFX
RRM2
UCK2
PBK
PKM
GAPDH
FAM207A
HMGA1
FAM76A
DCUN1D1
GCLC
SNN
FKBP4
MESD
B cells naive
B cells memory
Plasma cells
T cells CD8
T cells CD4 naive
T cells CD4 memory resting
T cells CD4 memory activated
T cells follicular helper
T cells regulatory (Tregs)
T cells gamma delta
NK cells resting
NK cells activated
Monocytes
Macrophages M0
Macrophages M1
Macrophages M2
Dendritic cells resting
Dendritic cells activated
Mast cells resting
Mast cells activated
Eosinophils
Neutrophils
Size
4
8
12
Color
−0.2
0.0
0.2
B
RiskScore
FAM76A
DCUN1D1
SNN
SHCBP1
MESD
ANLN
MELK
KPNA2
CEP55
PLK1
PKM
CENPK
AC099850.3
CENPE
KIF4A
CDKN3
PRC1
HMGA1
CKAP2L
MCM10
GTSE1
H2AFX
CCNB1
HMMR
CDK1
KIF23
CDC25C
RRM2
FAM207A
NEK2
DLGAP5
CENPH
NCAPG
HJURP
SGO1
KIF20A
FAM72A
NUF2
GAPDH
KNL1
E2F7
ARHGAP11A
KIF11
DEPDC1B
ECT2
FKBP4
PBK
KIF14
ASPM
UCK2
GCLC
HAVCR2
TNFRSF18
LAG3
CTLA4
CD274
TIGIT
TRAF6
CCL8
STAT5A
SDC1
SGPP2
IL2RA
IL1R1
CXCL10
CD4
CXCL11
FBLN7
CCL4
CD40
CCL2
IL1R2
IL10
CCL3
CCL20
CXCL9
CCL21
IGSF6
GFI1
CD163
CD200
CCL5
PDCD1
CD68
CCR5
CD38
ICOS
CD3D
CD8A
CCL18
CD3G
CSF2
CXCR3
CD5
SH2D1A
CD3E
CCL19
CR2
TNFRSF17
IRF4
FCER2
MS4A1
CXCL13
Size
10
20
30
40
50
Color
0.0
0.2
0.4
0.6
